# The copy number of Epstein-Barr virus latent genome correlates with the oncogenicity by the activation level of LMP1 and NF-κB

**DOI:** 10.18632/oncotarget.5708

**Published:** 2015-10-16

**Authors:** Lielian Zuo, Haibo Yu, Lingzhi Liu, Yunlian Tang, Hongzhuan Wu, Jing Yang, Meijuan Zhu, Shujuan Du, Lian Zhao, Li Cao, Guiyuan Li, Jianhong Lu

**Affiliations:** ^1^ Central Laboratory, Hunan Provincial Tumor Hospital, the Affiliated Tumor Hospital of Xiangya Medical School, Central South University, Changsha, Hunan 410013, China; ^2^ Cancer Research Institute, the Key Laboratory of Carcinogenesis and Cancer Invasion of the Chinese Ministry of Education, School of Basic Medical Science, Central South University, Changsha, Hunan 410078, China; ^3^ Department of Biological Sciences, Alabama State University, Montgomery, AL 36101, USA; ^4^ Department of Metabolism and Endocrinology, the Second Xiangya Hospital, Central South University, Changsha, Hunan 410011, China; ^5^ Current address: Cancer Research Institute, University of South China, Hengyang, Hunan 421001, China; ^6^ Current address: Department of Gastroenterology, the Third Xiangya Hospital, Central South University, Changsha, Hunan 410013, China

**Keywords:** Epstein–Barr virus, latent genome, copy number, latent membrane protein 1, oncogenicity

## Abstract

A tumor model that Epstein-Barr virus (EBV) latent infection facilitated the tumorigenicity was previously established using the Maxi-EBV system. In the present approach, EBV-lost cell clones demonstrated significantly decreased tumorigenesis. On the other hand, the LMP1 gene in Maxi-EBV genome was replaced by that of nasopharyngeal carcinoma origin. The resultant cell line, 293–1/NL showed much lower malignancy than the original 293-EBV. The result was opposite to our expectation. The change of 293 sublineage cells for EBV harboring also got similar result. To seek the underlying reason, the copy number of EBV genome in all the cell lines was detected. The result indicated that 293-EBV contained about 4.5-fold higher EBV copies than 293–1/NL did. Parallel EBV genomes led to relatively stable copies in different 293 sublineages, suggesting the viral genome structure is a factor for the sustainability of EBV's copy number. Moreover, the LMP1 transcription in high copy-containing cells showed abnormally high level. Furthermore, the main LMP1-driven pathway, transcription factor NF-κB, was highly activated in high-copy cells. Here we first manifest by experimental model that the copy number of EBV latent genome correlates with the viral pathogenesis, which depends on the activation level of LMP1 and NF-κB. Overall, both the presence and amount of EBV genome are crucial for the viral oncogenicity.

## INTRODUCTION

Epstein–Barr virus (EBV) is a ubiquitous human gammaherpesvirus that is the first confirmed oncogenic virus. It is highly associated with tumor development of both lymphoid and epithelial origins, including Burkitt's lymphomas and nasopharyngeal carcinoma (NPC) in which EBV genome can be found in almost all cells [1, 2]. EBV infection is usually latent with a minority of viral function proteins expressed, including latent membrane proteins such as LMP1 and LMP2A and Epstein–Barr nuclear antigen 1 (EBNA1) [1, 3, 4].

EBV is a complex virus due to its cancer association, special life cycle and large genome with more than 170kb of double strand DNA [5]. The etiological mechanism in cancer development remains largely to be understood. In order to study EBV's functions in the context of the whole viral genome, Dr. Hammerschmidt's group established a Maxi-EBV system which has been proven to be a useful tool in the study of the viral gene function, life cycle and genome maintenance [6–8]. The Maxi-EBV plasmid carries the complete EBV genome, selection markers in eukaryotic and prokaryotic cells, as well as an expression cassette for GFP. On the basis of this system, the establishment of EBV stably-infected cell lines has become feasible. By using the Maxi-EBV system and immortalized 293 epithelial cell line with low malignancy, we previously developed the cell line 293-EBV, and established a tumor model in nude mice [9]. This model makes it possible to analyze the tumorigenesis facilitated by the virus [9, 10]. With the selection resistance of hygromycin, the EBV genome-infected cell line also becomes a model for the study of EBV maintenance [9].

EBV-encoded LMP1 is known as the viral oncoprotein. We ever analyzed the sequence from 21 strains of full-lenth LMP1 genes of NPC origin (N-LMP1) [11]. Compared with that of the wildtype B95–8 strain, the sequence homology of these N-LMP1 genes ranged from 82.6% to 91.7%. The sequence variations made us to explore their possible pathogenesis difference of LMP1 in the Maxi-EBV genome. In the present study, the EBV stably-infected cells harboring N-LMP1 showed much lower malignant potential than that of 293-EBV. This result was out of our expectation, and urged us to further examine the underlying cause.

NPC cell lines primarily containing EBV genome are commonly observed to undergo EBV-lost process during passages *in vitro* [12, 13]. This phenomenon also happened to the stably-transfected cell line, 293-EBV, when cultured without selection pressure [9]. As demonstrated, the introduction of EBV genome exhibited enhanced proliferation and malignant potential [9]. In this study, to seek the possible difference of tumorigenesis between EBV positive and lost cells, we cloned these cells respectively, and compared their biological properties. The results showed the EBV-lost cells were restored to a low malignancy level similar to that of the donor 293 cells. Based on all results of this study, we are convinced that both sustained EBV infection and genome copy number at a relative high level are important for EBV to confer its pathogenesis. The transcription activation of LMP1 at high level is the direct result from EBV's high copy number, whereas the activation level of the main LMP1-driven NF-κB pathway is the consequent effect associated with the malignant potential level. To our knowledge, here we first verify by experimental model that EBV load in tumor cells correlates with its oncogenicity. The results imply that the viral load and LMP1-driven NF-κB are important factors involved in the cancer progression and should be considered in EBV-targeted therapy. Here we would present the whole “story” about the findings. The study would broader our understanding on the pathogenesis of EBV infection.

## RESULTS

### Loss of EBV genome resulted in decrease of tumorigenicity of the cells

**Figure 1:**
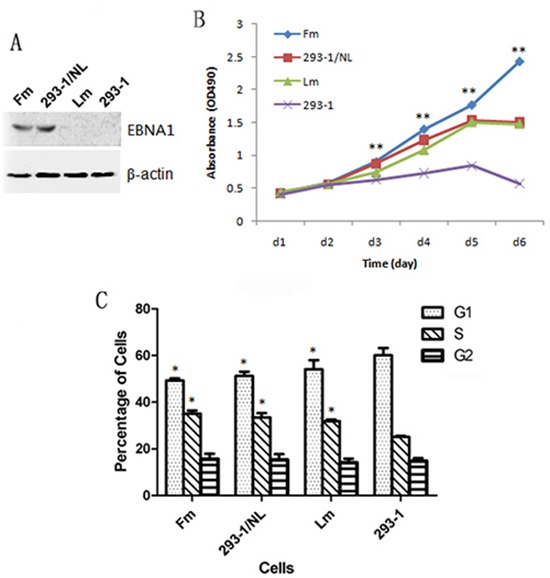
Effect of EBV genome loss or N-LMP1 replacement in EBV genome on the growth ability of the cells **A.** The expression detection of EBNA1 in the cells by western blotting. **B.** Growth curve assayed by MTT analysis. **Increased growth ability with extremely significant differences (*p* < 0.01) for: Fm *versus* Lm or 293–1 cells (at 3–6 d); Lm *versus* 293–1 cells (at 4–6 d); 293–1/NL *versus* 293–1 cells (at 3–6 d). **C.** Alteration in cell cycle distribution of EBV-infected cells. *The G1 and S phase of the cells, Fm, 293–1/NL and Lm, showed significant difference compared with 293–1 cells (*p* < 0.05). For (B) and (C), the data corresponded to the mean values of three independent experiments.

**Figure 2:**
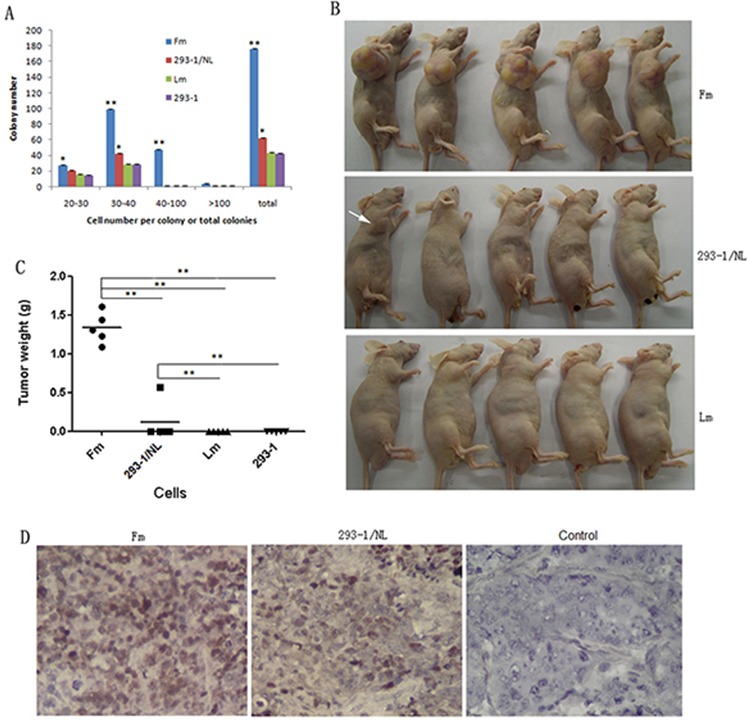
Colony formation in soft agar and tumor development in nude mice for the cells **A.** Colony number for each cell line. The colonies were counted manually according to the cell number range. **p < 0.01, *p < 0.05 compared with Lm or 293–1 cells. **B.** Tumor formation in nude mice (*n* = 5) at 7 weeks post-injection. For the 293–1/NL group, only one tumor formed as indicated (arrow). The 293–1 control group was used to an extended observation for tumor formation, and thus the mice are not shown here. **C.** Tumor weight variation for each group. **p < 0.01. **D.** EBV genome detection in the tumors by ISH for EBER1. Control, no EBER1 probe added. Original magnification, × 400.

During the culture of 293-EBV *in vitro*, EBV genome was found to become lost from the cells gradually when without selection pressure. In order to know whether there would be changes in biological properties for the EBV-lost cells, clones of GFP-positive and GFP-negative were selected. They were identified by the detection of the gene transcription and protein expression of EBNA1. Here, the EBV genome-lost cell line, Lm, a mixture of 10 EBNA-negative clones showed a relative high ability of proliferation compared with the original 293–1 cells (Figure 1). Whereas the results of colony formation in soft agar and tumor formation in nude mice showed that the malignant potential decreased significantly when compared with the EBV-positive cell line, Fm (Figure 2). The result suggested that EBV-lost cells were restored to a low malignancy level as the donor cells showed.

### The cell line 293–1/NL with the replacement of N-LMP1 in EBV genome showed much lower tumorigenicity than the original 293-EBV cell line

**Figure 3:**
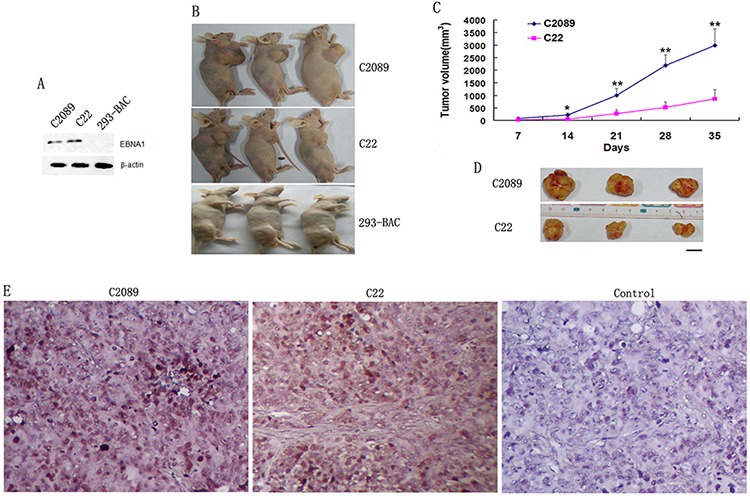
Tumor formation for the EBV stably-transfected cells after the change of donor cell line (293–2) **A.** EBNA1 expression in the cells detected by western blotting. **B.** Tumor formation in nude mice (*n* = 3) at day 35 post-injection. **C.** The growth curve of the tumors for C2089 and C22 cells. **D.** Tumors formed by C2089 and C22 cells at day 35. Bar, 10 mm. **E.** EBV genome detection in the tumors by ISH for EBER1. Control, no EBER1 probe added. Original magnification, × 200.

In order to study the function of N-LMP1 in the EBV genome, one full-length N-LMP1 gene was chosen to replace the B-LMP1 gene in Maxi-EBV through homologous recombination technique [14]. All the generated cell lines in this study are designated as in Table 1. Since N-LMP1 was proposed to possess higher malignancy than B-LMP1 in single gene analysis [15], we had predicted that N-LMP1 in genome analysis might also show higher tumorigenicity. In this study, the result was out-of-expectation. The cell line 293–1/NL showed much lower ability in colony and tumor formation tests than the cell line, Fm, which contained the Maxi-EBV genome (Figure 2 and Figure 3). Yet, 293–1/NL showed increased proliferation and tumorigenicity compared with the donor cell line, 293–1 (Figure 1, Figure 2 and Table 2).

**Table 1:** Origin of the cell lines

Cell lines	293-EBV	Fm	Lm	293–1/NL	C2089	C22	293–BAC	C2089/neo
Derived from	293–1	293-EBV (293–1)	293-EBV (293–1)	293–1	293–2	293–2	293–2	293–2
EBV genome	full	full	lost	full with NPC-LMP1	full	full with NPC-LMP1	None/removed	full
Selection resistance	hyg^r^	hyg^r^	None	hyg^r^	hyg^r^	hyg^r^	hyg^r^	neo^r^
GFP expression	+	+	–	+	+	+	+	+

Hyg, hygromycin; neo, neomycin. +, positive; –, negative.

**Table 2:** Tumor formation of the cell lines in nude mice

Cells	Frequency	Day when first detected	Average tumor weight at week 7 (g)
Fm	5/5	15	1.54
293–1/NL	1/5	47	0.52
Lm	0/5	-	-
293–1	0/5	-	-
Culture medium of Fm	0/4	-	-
Cell lysis of Fm	0/5	-	-

2 × 10^6^ cells or the supernatant and cell lysis were injected subcutaneously. Tumors developed at injection site and were first detected by palpation.

### The change of donor 293 sub-lineage cells demonstrated similar result in tumorigenicity for the derived cell line harboring N-LMP1 *versus* B-LMP1

Though the cell line 293 appears sufficiently stable during cultivation [16], it may exhibit diverse malignancy potential. This genetic alteration might be due to different patterns of continuous *in vitro* passage pressure, for example, exceeding 52 passages in one thaw-freezing cycle [17]. The sub-lineage 293–2 has a higher ability to form tumors in nude mice than 293–1 (3/6 *versus* 1/5 within 7 weeks at 4 × 10^6^ of injected cells). In order to verify above result for N-LMP1 in genome analysis, these two EBV genomes containing B-LMP1 or N-LMP1 were introduced into 293–2 cells respectively. Two cell lines, C2089 and C22, were accordingly produced after hygromycin selection process (Table 1). As shown in the tumor formation test (Figure 3), from the tumor growth sizes within 5 weeks, C22 also showed lower malignancy than C2089. The result showed similar malignancy difference between C2089 and C22 cells as Fm and 293–1/NL did.

### The copy number of EBV genome in tumor cells corresponded to the severity of tumorigenic potential

**Figure 4:**
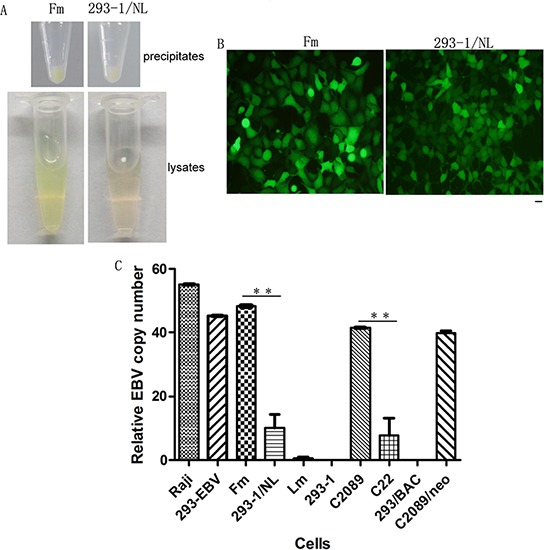
Difference of the EBV genome number in the cells **A.** The color difference between the precipitates and lysates of Fm and 293–1/NL cells. About 5 × 10^6^ cells of these two cell lines were used respectively. **B.** Fm and 293–1/NL cells observed under a fluorescent microscope. Bar, 100 μm. **C.** The relative copy number of EBV genome in the cell lines. The copy number of Raji was standardized to 55 per cell. **p < 0.01.

The unexpected results in EBV genome analysis firstly made us suspect the donor cell difference from the reported Rhex-1 [15]. We subsequently established stably-transfected cell lines expressing N-LMP1 or B-LMP1 single gene using 293–2 cells, and they did not show obvious difference in tumorigenicity in nude mice (data not shown). This prompted us to further explore the underlying cause. During the cell freezing, a fact was noticed that the cell precipitation color of Fm/293-EBV and 293–1/NL was different (Figure 4A). The lysis suspension of Fm showed deeper green than 293–1/NL (Figure 4A). While no obvious color difference at monolayer for these two cell lines was noticed under a fluorescent microscopy (Figure 4B). Since GFP is linked to the EBV genome, the color difference suggested their different amount of EBV genome. We then thought of determining the EBV copy number in each cell line. As the results showed in Figure 4C, the cells, Fm possessed about 4.5-fold higher EBV copy number than that in 293–1/NL. For the same EBV genome (e.g. in Fm and C2089), no matter what sub-lineage cells (293–1 or 293–2) they were harbored in, they were at almost identical level of copy number. This was able to be seen with the cell lines, Fm, C2089 and C2089/neo (Figure 4C). The cells 293–1/NL and C22 with the same EBV genome also contained EBV copies at low level identically.

The result of EBV copy number difference corresponded to the tumorigenicity difference showed above. The cells (Fm and C2089) containing higher EBV copy number exhibited higher malignancy in nude mice. To see whether equal total EBV copies might have similar tumorigenicity, 4.5 × 10^6^ of 293–1/NL cells and 1 × 10^6^ of Fm cells per mouse respectively were used to compare their tumor formation ability. As it shown in Table 3, the 293–1/NL group still had lower tumorigenicity.

**Table 3:** Tumor formation in nude mice with equal EBV copies in cells

Cells	Cell number per mouse	Tumor Frequency	Days when first detected	Average tumor weight at week 7 (g)
Fm	1 × 10^6^	3/5	19, 22, 28	1.12
293–1/NL	4.5 × 10^6^	2/5	34, 42	0.65

Under the same EBV copy number level neglecting of the cell number, Fm cells also showed higher malignancy than 293–1/NL.

### High EBV copy number resulted in high activation level of LMP1 transcription and LMP1-driven NF-κB pathway

**Figure 5:**
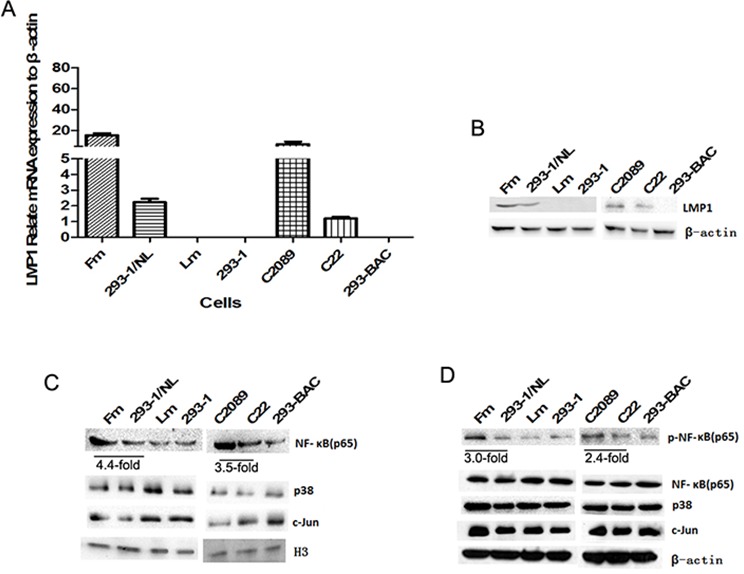
The detection of LMP1 and LMP1-driven pathways **A.** The transcription of LMP1 in the cells detected by real-time qPCR. **B.** The expression of LMP1 in the cells by western blotting assay. **C.** The expression of NF-κB (p65), p38 and c-Jun in nuclear. Histone 3 (H3) was used as a control for nuclear proteins. **D.** The expression of p-NF-κB (p65), NF-κB (p65), p38 and c-Jun in cellular general proteins.

The expression of the oncoprotein LMP1 in the cells was detected (Figure 5A and 5B). The result of mRNA level by qPCR showed about 10 to 18-fold higher in Fm and C2089 cells than in 293–1/NL and C22 cells. Compared with the copy number difference (4.5-fold), the LMP1 transcription with B-LMP1 is somewhat abnormally activated.

LMP1 activates 3 main pathways, NF-κB, p38/MAPK and AP1 (c-Jun), and NF-κB is the most important [18–20]. Rel A (p65) is the major component of NF-κB. In this study, the expression of these key molecules in the cells was detected. The NF-κB pathway was significantly activated (see nuclear and phospharylated NF-κB) (Figure 5C and 5D). Compared with 293–1/NL, Fm had about 4.4-fold nuclear NF-κB and 3.5-fold phosphorylated NF-κB. This perfectly corresponded to their difference in EBV genome copy number. Regarding p38 and c-Jun pathways, Fm cells gained a little more at their general expression level compared with 293–1/NL cells (Figure 5D). At the in-nucleus expression level, there was also no significant difference for p38 or c-Jun between different cells (Figure 5C), suggesting that these two pathways did not act as markedly as NF-κB did.

## DISCUSSION

To explore the causative role for EBV latent infection especially in epithelia cancers, cell and tumor models are important, but not satisfactory currently. The difficulty lies in several aspects of problem. One is that EBV is not able to infect epithelial cells directly *in vitro* for the lack of receptor CD21 on the cell membrane [1, 21]. Another is that EBV is not able to be stably maintained in the cells during culture [9, 12]. The come-out of herpesvirus genome based on BAC system such as the Maxi-EBV system with a selection resistance in cells has allowed us to develop models for studying the virus itself [6, 9]. It is superior studying the complex virus as EBV in the context of the whole genome to single gene expression analysis. As tumor viruses are usually not complete carcinogens [22], it is difficult to establish a tumor model in epithelial cells transformed by virus only. Using the Maxi-EBV system and 293 epithelial cell line with low malignancy, we previously established the cell line 293-EBV, which displayed EBV's role in facilitating the cellular malignant potential [9]. With the hygromycin selection in culture, it also becomes a useful tool to study the viral maintenance in host cells. In the present approach, based on this system, we established a series of cell lines and the results showed the persistence of EBV genome in the cells is important for the tumorigenesis. The results also led to the serendipity that high copy number of EBV correlate with highly enhanced malignancy potential induced by EBV through high activation of LMP1 and LMP1-driven NF-κB pathway.

It is widely shared that EBV-positive epithelial cells lose EBV genome during passages *in vitro* [9, 12, 13]. This is even true for EBV-immortalized lymphoma cells which have the membrane receptor CD21 for the viral re-infection in culture [23]. This phenomenon has been of interest. In this study, we cloned the EBV-positive and EBV-lost cells, the result showed EBV-lost cells (Lm) still showed higher proliferation ability than the donor 293–1 cells (Figure 1), probably due to the viral infection hit. While the tumorigenicity tests showed Lm had a restored malignancy to a level as the origin 293–1 cells (Figure 1 and 2). The results demonstrate that the persistence of EBV genome in the cells is important for the enhanced tumorigenesis of the infected cells. This is a reverse confirmation for the enhanced tumorigenesis by EBV as in the 293-EBV cells. It is consistent with what described in the Burkitt's lymphoma cell line Akata [23]. It has been also conversely shown that the re-introduction of EBV in EBV-lost NPC cells enhances the tumorigenicity [12]. Based on our experiments, we have previously proposed a cell-to-cell mechanism for the effective maintenance of EBV genome in cells [9]. As a fact, EBV is not so easy to be lost from NPC tissue cells because the virus can be found in almost all the cancer cells [1–3]. Overall, it seems that the loss of EBV during culture is more in a compulsive way than active run-away. The mechanism of how the virus is “driven away” from the host cell remains to be explored.

As described previously [11], there is a sequence difference in LMP1 gene between B95–8 and NPC strains from Hunan province, which locates in southern China and has a high NPC incidence rate. We were to seek the pathogenesis difference of LMP1 between B95–8 and NPC strains using the Maxi-EBV system. Thus the full-length of B-LMP1 was replaced precisely by N-LMP1 through the method of homology recombination in *E.coli* [14]. Other's research has suggested that N-LMP1 expressed in Rhex-1 cells had higher tumorigenicity in SCID mice than B-LMP1 [15]. Therefore, we hoped to establish a tumor model enhanced by EBV with N-LMP1 using our genome analysis system. The result was on the contrary of our expectation. The cell line 293–1/NL showed low tumorigenicity which was close to that of the origin 293–1 cells (Figure 2 and Table 2). Similar result was obtained with the change of donor 293–2 sublineage (Figure 3). Owing to no different tumorigenicity in nude mice between B-LMP1and N-LMP1 in single gene expression in 293–2 cells (data not shown), this result ever became puzzling to us. The study had been standing still until the color difference of cell precipitation was noticed (Figure 4A). EBV copy number was then focused on. As shown in Figure 4C, high EBV copy number in the cells corresponded to the high tumorigenicity. As a whole, parallel EBV genomes had relatively stable copies in the 293 cell line (e.g. wt B95–8 genome in 293-EBV, C2089 and C2089/neo cells; LMP1-mut genome in 293– 1/NL and C22). The result suggests that the viral genome structure is a factor for EBV to sustain at a relatively constant level of copy number in host cell. Regarding to the reason why EBV may sustain stable copy number in host cells, a series factors should be considered. Firstly, in this study, the N-LMP1 was embedded into the EBV genome of B95–8 strain which was not the original genome for the compatibility of N-LMP1. As described, EBV-encoded EBNA1itself can regulate the expression of LMP1 and host genes by acting as a transcription factor [24, 25]. Whether there is some other internal regulation mechanism remains unknown. Secondly, it has been recently showed that some host factors are involved in the regulation of EBV copy number in immortalized lymphocyte lines [26]. In the present approach, we used the same donor cell line (293) for EBV genome harboring, and the change of selection resistance gene (for C2089/neo cells) did not have influence on the EBV copy number (Figure 4C). Whether the different origin and expression level of LMP1would possibly result in different expression of some host genes, a further study needs to be done. For our part, the relatively stable copy number of EBV should be resulted from a balance between the host and virus factors.

Though the cell line 293 is not particularly relevant for EBV infection, we have shown that the 293-EBV tumor model in nude mice is useful for EBV oncogenicity analysis [9, 10]. Nevertheless, some conditions should be limited. For example, suitable range of cell number, e.g. 1 × 10^6^ to 4 × 10^6^ cells, and suitable range of observation time (e.g. 5 to 7 weeks) are included [9]. To our experience, the higher malignant potential for the donor 293 cells, the less cells and shorter observation time should be designed in the experiment. Beyond these limits, the tumor growth for both the EBV+ and the EBV- cells could become out-of-control due to the malignant potential of the donor cells themselves. We have also shown that for the same cell line, 293-EBV, its tumorigenicity is dose-dependent [9]. In view of the difference of EBV copy number and the dose-dependence in tumor formation, we used the same total EBV copies neglecting of the cell number in tumor formation. As shown in Table 3, the group of 293-EBV still exhibited higher malignancy than the 293–1/NL group. The result implies that the pathogenesis of EBV is not a simple additive effect by copy number. The copy number in a single cell or average level seems more paramount than general level.

LMP1 is an important functional protein in EBV oncogenicity [1–3, 27]. In this study, the abnormally high activation of LMP1transcription corresponded to high EBV copies in the cell lines (Figure 4C). This suggests that the effect of LMP1 activation is not in a way of simple proportional addition as an effect to the EBV copy number. Nevertheless, the EBV genome copy number in the cells is an important factor for the pathogenesis.

The C-terminal activating region (CTAR) domains of LMP1 activate three pathways, including the transcription factor NF-κB, p38/MAPK and c-Jun/AP-1 [18–20]. We detected the activation of the three pathways. Among them, NF-κB (p65) was at high levels of nuclear expression and phosphorylation (Figure 5C and 5D) in high-copy cells. NF-κB activation is a central event regulating inflammation and cancer development [28, 29]. In our previous study on EBV-LMP1 function, the NF-κB was also a key pathway [10]. It is noticeable that the NF-κB activation level perfectly corresponds to the level of EBV copy number in the same cells.

In this study, we present the first evidence by experimental model that the copy number of EBV is an important factor involved in the viral pathogenesis. We thus speculate that one cell with very low EBV copy number might not be transformed easily and develop a cancer. This may partly explain the fact that the ubiquitous virus is associated with only a minority of cancer. It has been noticed that EBV load in tumor tissues or blood is associated with the clinical progression and prognosis in both lymphoma and NPC [30–33]. Our result verifies this association. We also emphasize the importance to measure the level of gene expression or copy number in the virus study instead of only concerning “with and without”. In addition, the identification of IgA antibody to EBV VCA (viral capsid antigen) is associated with EBV infection in NPC clinically [2, 34, 35]. However, in immunocompromised patients, serologic data do not always represent clinical data. On this occasion, quantitative analysis of EBV copy number might become a better interpreter for EBV-associated diseases.

Taken together, the persistent latent infection of EBV genome in tumor cells is confirmed again an important factor for the tumorigenicity. Moreover, relative high copy number of EBV genome correlates with the development of cancer and should be considered into the cancer etiology. Although the precise role of EBV in the carcinogenic process remains to be fully understood, the oncoprotein LMP1 is a throughout crucial factor for the viral oncogenicity. The viral load and LMP1-driven NF-κB are important factors for the understanding of disease progression and EBV-targeted therapy.

## MATERIALS AND METHODS

### Plasmids, cell lines and antibodies

The plasmid p2089 (Maxi-EBV) which contains the complete EBV genome of B95–8 strain was kindly provided by Dr. W Hammerschmidt [6]. The human embryonic kidney (HEK293, 293) epithelial cell line was grown in Dulbecco's modified Eagle's medium (DMEM)(Gibco, California, USA) complemented with 10% fetal calf serum. Here we used 2 sub-lineages of 293 (293–1, 293–2, both ATCC origin) coming from our lab and another group in our institute respectively. There is a difference in malignant potential on 4-week-old nude mice due to their different passage patterns. They could made 1/5 (for 293–1) or 3/6 (for 293–2) tumor formation in nude mice within 7 weeks when injected with 4 × 10^6^ of cells [9, 36]. Cell line 293-BAC was established by stably transfected with plasmid pM-BAC in 293–2 cells [37]. The plasmid pM-BAC was constructed as described by deleting the whole EBV genome in p2089, leaving only the cassette of selection gene and BAC F-factor [37]. It could be used as a control for p2089. The LMP1 and EBNA1 monoclonal antibodies (mAb) were products of Dako Cytomation (DAKO Lifetech, Glostrup, Denmark) and Santa Cruz (Biotech., Delaware, USA) respectively. Other antibodies are anti-: p-NF-κB (p65) (rabbit mAb, CST, IL, USA), NF-κB (rabbit mAb, Millipore, MA, USA), c-Jun rabbit mAb (Epitomics, CA, USA) and p38/MAPK (Anbobio, CA, USA). The β-actin antibody (Proteintech, IL, USA) and anti-Histone 3 (H3) antibody (Sigma-Alorich, MO, USA) were used as loading controls.

### Generation of cell lines stably transfected with plasmids containing EBV genome

One full-length N-LMP1 gene (NPC4, GenBank accession No. EF419187) was chosen for this study. It had a homology of 88.8% compared with B-LMP1 [11]. By homology recombination technique as described previously, B-LMP1 gene was precisely replaced with N-LMP1 gene in the plasmid p2089 [14, 37]. The resultant plasmid was designated as p2089/NL. By using the same technique, the hygromycin resistance gene in p2089 was replaced by neomycin gene, resulting in p2089/neo plasmid. To generate cell lines, the plasmids, p2089/NL, p2089/neo were transfected into 293–1 and 293–2 cells respectively according to the protocol of the production of 293-EBV [9]. After hygromycin (Roche Diagnostics GmbH, Mannhein, Germany) or G418 (Gibco, California, USA) selection, pool clones were obtained. All the cell lines were cultured as 293 cells and made to 100% GFP-positive by resistance selection before the biological property assays or other detection tests.

### Cloning of EBV positive and negative cells from 293-EBV

The EBV genome in 293-EBV could be lost during passages when cultured without selection pressure especially at low density [9]. The 293-EBV cells with about 40% of GFP negative (GFP-) were diluted and planted onto a six-well plate at about 10 cells per well. GFP positive (GFP+) and GFP- clones were selected and plated into 12-well plates respectively. Clones were amplified and identified for EBNA1 gene or protein expression by PCR and western-blotting assays. Ten GFP+ and EBNA+ clones were mixed to form the Fm cell line, and 10 GFP−/EBNA− clones to form the Lm cell line.

### MTT assay and cell cycle analysis

MTT assay was performed to measure the cell proliferation as described [9]. Briefly, the cells were cultured in 96-well plates to the time points as indicated. The cells were then incubated with 20 μl of 5 mg/ml MTT (Sigma, Missouri, USA) in complete medium for 4 h and dissolved in dimethyl sulfoxide (MP Biomedicals, California, USA). Absorbance at 490 nm was measured. For cell cycle analysis, the cells were trypsized and collected, then washed with PBS, fixed in 70% cold ethanol. The cells were subject to cell cycle analysis performed on FACScan Flow Cytometer using CellQuest software (BD Biosciences, BJ, USA). The experiments were repeated for three times.

### Colony formation in soft agar

This assay is a method for evaluating the ability of individual cell lines to grow in an anchorage-independent manner. Culture medium of 3ml containing 0.5% agar (Life Science, Tokyo, Japan) and 10% fetal bovine serum was plated in six-well plates and allowed to solidify, creating a bottom layer. The cell suspension in 2 ml culture medium was combined with 0.3% agar, and plated onto the top of the bottom layer. The plates were examined twice per week till 30 days. The colonies were counted manually after 3 weeks. The experiment was repeated for three times.

### Tumor formation in nude mice

Cells of the amount as indicated were suspended in 200 ul growth medium and subcutaneously injected into the 4-week-old BALB/c nude mice (Slaccas Com., Shanghai, China). The mice were monitored and measured for the appearance and growth rate of tumors. Cell growth supernatant and cell lysates correspondingly were used for negative control. Preparing for histological examination and EBV detection, tumor tissues were dissected, fixed in 10% buffered formaldehyde, and embedded in paraffin.

### Western blotting analysis

Western Blotting was performed with a standard protocol. The general cell protein or nuclear proteins were extracted respectively. Proteins were separated on 10% SDS-polyacrylamide gels, electrophoretically transferred to polyvinylidene difluoride membranes (Millipore, Danvers, MA, USA), and detected using a mouse monoclonal antibody for anti-EBNA1 (Santa Cruz Biotech., Delaware, USA) or anti-LMP1 (DAKO, Glostrup, Denmark). The β-actin antibody (Proteintech^TM^, Chicago, USA) was used as a loading control. The detection was performed on the ChemiDoc XRS+ Molecular Imager (Bio-Rad) using a Western Bright ECL kit (Advansta, California, USA). The Image J 2X software was used for the Gray-Scale quantification of the bands.

### *In situ* hybridization for EBV detection

Oligonucleotide probe of EBER1 was digoxigenin-labeled at the 3′ terminus. The tumor specimens were paraffin-embedded and sectioned. ISH were performed according to the manufacture's instruction of the detection kit (Boster Inc., Wuhan, China).

### RT and real-time quantitative PCR (RT-qPCR)

Total RNA was isolated from the cells using TRIzol reagent. Protocol of a reverse transcription reaction was performed using the First Strand cDNA Synthesis Kit (Thermoscientific, MA, USA), and qPCR was performed using a Maxima SYBR Green qPCR Master Mix (2 ×) kit (Thermoscientific, MA, USA) according to the manufacturer's instructions. A CFX Multicolor Detection System (Bio-Rad) was employed for the detection. The primers in qPCR reactions for LMP1 were as: (forward, 5′-TGAACACCACCACGATGACT-3′; reverse, 5′-GTGCGCCTAGGTTTTGAGAG-3′), and for β-actin were as: (forward, 5-GCATCCCCCAAAGTTCACAA-3′; reverse, 5′-AGGACTGGGCCATTCTCCTT-3′). The following program was performed using a two-step cycling protocol : an initial denaturation at 95°C for 10 min, followed by 40 cycles of 95°C for 15s, 60°C for 60 s. Three parallel repeats were performed for each sample in each experiment, and the results were expressed as the mean of three independent experiments.

### Detection of EBV copy number

General DNA was extracted from cells by using the General AllGen Kit (CWBio, Hunan, China) and quantified. Relative EBV copy number was detected according to the manufacturer's instructions of Epstein-Barr virus DNA Quantitative Fluorescence Diagnostic Kit (Sansure Biotech, Hunan, China). Specific PCR-fluorescence probe and a pair of specific primers for EBV conserved regions were provided in the kit. Real-time qPCR was performed. Two channels were detected. HEX channel was applied to detect the fluorescence of the internal standards to avoid false-negative results, requesting every sample to run an “s” curve which is also a basic request in real-time qPCR. The standard curve (R2 ≥ 0.98) was drawn according to the fluorescence signal changes (FAM channel) of quantitative reference substances (A/B/C/D), EBV-positive and EBV-negative controls. The EBV copy number concentration (copies/ml) of the samples was then calculated according to the standard curve. In the comparison of the relative copy number, the copy number of Raji which was standardized to 55 per cell, for Raji is accepted to contain 50–60 EBV latent genomes per cell [38]. The experiment was repeated for three times.

### Statistical analysis

Statistical analysis was determined by one-way analysis of variance (ANOVA) test using the SPSS, version 11.0, program.
